# Retinal Layer and Choroidal Changes in Deep and Scuba Divers: Evidence of Pachychoroid Spectrum-Like Findings

**DOI:** 10.1155/2024/1600148

**Published:** 2024-11-15

**Authors:** Nur Demir, Belma Kayhan, Mertan Acar, Sukru Sevincli, Murat Sonmez

**Affiliations:** ^1^Ophthalmology Department, Sultan Abdulhamid Han Training and Research Hospital, University of Health Sciences, Istanbul, Türkiye; ^2^Underwater and Hyperbaric Medicine Department, Marine Rescue and Underwater Command, Istanbul, Türkiye

**Keywords:** choroid, deep diving, pachychoroid, retinal pigment epithelium, scuba diving

## Abstract

**Purpose:** Diving is an intense physical activity under hyperbaric and hyperoxic conditions. The aim of this study is to evaluate the long-term effects of diving on the thicknesses of retinal layers and retinal anatomy in professional deep and scuba divers.

**Methods:** The study included 52 eyes of deep divers who dive to depths of more than 130 feet (ft), 49 eyes of scuba divers who dive up to 130 ft, and 66 eyes of the control group, consisting of nondiving but regularly exercising males. Measurements of macular retinal layer thicknesses, peripapillary nerve fiber layer thickness, subfoveal choroidal thickness, and peripheral retinal examinations with scleral indentation were performed and statistically compared between the groups.

**Results:** The mean diving duration was 455.00 ± 318.88 h in deep divers and 451.67 ± 281.10 h in scuba divers. The retinal pigment epithelium (RPE) was statistically significantly thicker in deep divers than in scuba divers and the control group on the 3 mm ring of the Early Treatment Diabetic Retinopathy Study grid. Subfoveal choroidal thickness was significantly thicker in deep divers than in scuba divers (*p* < 0.05). RPE abnormalities showed a significant increase in both the deep and scuba diver groups (*p*=0.01).

**Conclusion:** An increased thickening of the subfoveal choroid and RPE, resembling pachychoroid pigment epitheliopathy, was detected in deep divers over a long-term duration.

## 1. Introduction

Professional divers are trained as deep divers and scuba divers. Deep divers dive to depths of more than 40 m of seawater (msw) (130 feet) [1]. Divers perform their physical activities in an environment characterized by hyperoxia, increased ambient pressure, and cold temperatures [2].

Ambient pressure increases by one absolute pressure in the atmosphere (ATA) with every 10 msw of diving. The solubility of all gases increases proportionally to the partial pressure according to Henry's Law [3]. With the increase in ambient pressure, the amount of gas dissolving into the liquid also increases [4, 5]. Increased oxidative stress, decreased antioxidant capacity, circulating bubbles, and peripheral vasoconstriction have been studied as responsible factors for endothelial dysfunction and decompression sickness (DCS) in divers [6–9].

Diving also has shown to induce ocular damage [10–12]. Ocular barotrauma, DCS, central retinal artery occlusions, central serous chorioretinopathy (CSR), retinal pigment epithelium (RPE) abnormalities, altered color/contrast sensitivity, and visual field scotomas have been reported in diving studies [10, 11]. Divers have a higher incidence of RPE abnormalities [12]. Retinal damage is found to increase with long-term diving [13]. However, very few studies have evaluated the retina in professional divers, and there are no studies comparing the retinas of deep and scuba divers. This study aims to evaluate the long-term effects of diving on the thickness of retinal layers and retinal anatomy in professional deep and scuba divers.

## 2. Methods

The study was conducted in accordance with the Declaration of Helsinki and was approved by the ethics committee. All participants signed written informed consent.

**Figure 1:**
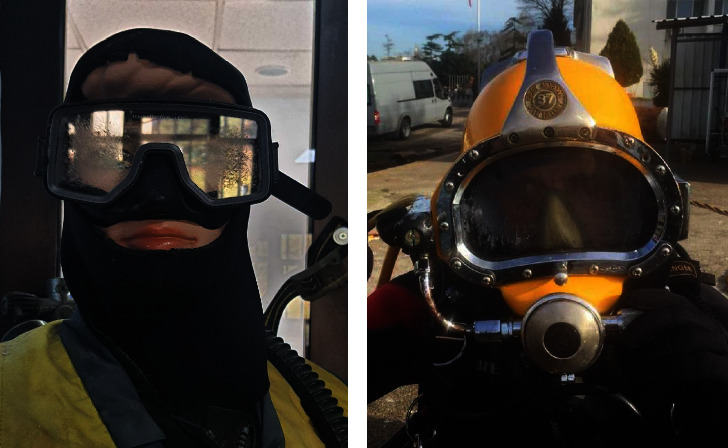
Divers' equipment. (a) A scuba diver with a half-face mask and (b) a deep diver with a helmet (supplied air diving system).

Two study groups and a control group were formed to compare the data. All participants were males of the same ethnicity. The scuba diver group consisted of cases who dived only up to 130 ft, with a half-face mask (Figure 1(a)). The deep diver group consisted of cases who dived only between 130 and 300 ft, using a helmet with a surface-supplied diving system (SSDS) mechanism (Figure 1(b)). A control group was formed from the eyes of male participants who did not dive but performed regular conditioning training.

All participants ceased diving and exercise 1 week before participation in the study. The medical records of the patients were carefully examined for gas embolism, DCS, and CSR. Participants were also asked about symptoms that might be related to these diseases, such as abnormal fatigue, joint and muscle pain, dizziness and numbness, shortness of breath, itchy skin and rashes, confusion, coughing up frothy and bloody phlegm, unconsciousness, tremors, and amnesia.

The body mass index of all participants was between 22.5 kg/m^2^ and 28.8 kg/m^2^. All of them underwent annual health checks and were found to be healthy based on a comprehensive yearly systemic evaluation. Refractive error was within +1.00/−1.00 D. Exclusion criteria included a history of any intraocular surgery, optic neuropathy, hypertension, diabetes, vascular disease, anterior or posterior segment inflammation, laser photocoagulation, and glaucoma. Participants in the diver and control groups with any preexisting ocular/retinal disease were excluded from the study.

Participants were examined for best-corrected visual acuity (BCVA) using the Early Treatment Diabetic Retinopathy Study (ETDRS) chart, intraocular pressure (IOP), and the slit-lamp anterior segment. A dilated fundus examination was performed using a scleral compressor to thoroughly examine the periphery.

The retinal and choroidal layers were measured by using spectral-domain optical coherence tomography (SD-OCT) (Version 1.10.4.0, Software_V6.16.2, Heidelberg Engineering, Heidelberg, Germany) with pupil dilation. For retinal measurements, images were taken in raster-scan mode on the OCT. ETDRS grid was centered on the foveola. Total retinal thickness, and the thicknesses of the retinal nerve fiber layer (RNFL), ganglion cell layer (GCL), inner plexiform layer (IPL), inner nuclear layer (INL), outer plexiform layer (OPL), outer nuclear layer (ONL), and RPE were segmented and analyzed in the superior, inferior, nasal, and temporal subfields of the inner and outer areas of the ring. Structural changes in the retina and choroid were evaluated in each sectional scan using these images. Any detected structural changes were recorded. Peripapillary RNFL was also evaluated with SD-OCT in the superior, inferior, temporal, and nasal quadrants.

Choroidal thickness (CT) was measured 500 μm temporally and nasally from the fovea, as well as in the subfoveal area, using EDI-OCT scans. The mean CT was calculated as the average of the temporal, nasal, and subfoveal thicknesses. Both subfoveal measurements and the mean CT value were used in statistical comparisons. All SD-OCT measurements were taken by one technician. One ophthalmologist examined all patients and recorded the data. Retinal layers were segmented automatically, and segmentation errors were corrected manually.

### 2.1. Statistical Analysis

Statistical Package for Social Sciences (SPSS 20.0, Chicago, IL) was used for statistical analysis and interpretation of the data. Continuous variables of descriptive statistical methods were reported as the mean and standard deviation. Categorical variables of descriptive variables were reported as percentage. The compatibility of the quantitative data to normal distribution was analyzed with Shapiro–Wilk test. One-way analysis of variance (ANOVA) and independent samples *t*-test were applied to test for comparison of normally distributed variables. Categorical variables were analyzed by Pearson chi-square test. *p* values of less than 0.05 were considered statistically significant.

## 3. Results

Fifty-two eyes of deep divers and forty-nine eyes of scuba divers constituted the two study groups. The control group consisted of 66 eyes. Only one eye of each participant was evaluated. There were no significant differences in the mean age, BCVA, refractive values, or diving hours between the groups (Table 1). The mean diving duration was 10.7 years in the deep diver group and 13.9 years in the scuba diver group, with the median diving duration being 8 years (2–30 years) in the deep divers and 12 years (2–31 years) in the scuba divers.

None of the participants had a history of DCS or CSC nor any symptoms related to these conditions.

None of the retinal layers or the full retina in the central 1 mm ring of the ETDRS grid showed a statistically significant difference in thickness between the groups (Table 2). The RPE layer was statistically significantly thicker in the deep diver group than both the scuba diver group and the control group in the inner 3 mm ring of the ETDRS grid (*p* < 0.05) (Table 3). The RPE layer was also thicker in the deep divers than in the control group in the inner 1 mm ring and outer 6 mm ring of the ETDRS grid, but this difference was statistically insignificant (*p* > 0.05) (Table 2 and Table 4).

None of the retinal layers or the full retina in the outer 6 mm ring of the ETDRS grid showed a statistically significant difference in thickness between the groups (Table 4).

**Figure 2:**
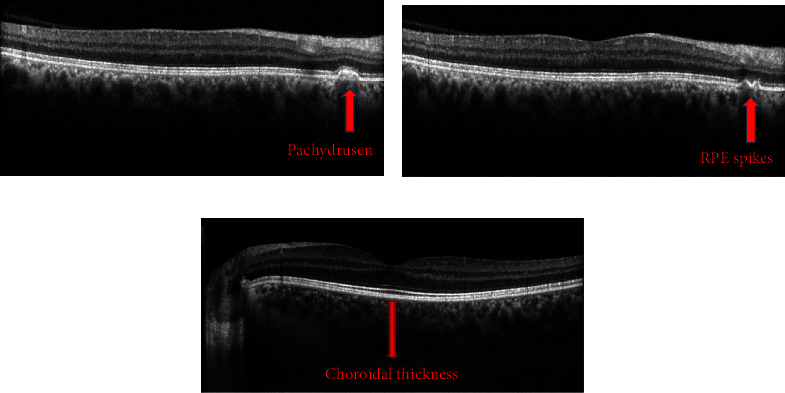
Spectral-domain optical coherence tomography scans of the eyes with retinal pigment epithelium (RPE) abnormalities and choroid in deep divers. (a) Pachydrusen, (b) hyper-reflective spike of RPE, and (c) EDI-OCT image of thickened choroid and pachyvessels.

RPE abnormalities (pachydrusen, RPE thickening, and RPE spike) demonstrated a significant increase in the study groups; RPE abnormalities, including pachydrusen, RPE thickening, and RPE spikes, showed a significant increase across the study groups: 9 eyes in the deep divers (3 with pachydrusen, 5 with RPE thickening, and 1 with an RPE spike) accounting for 17.3%, 6 eyes in the scuba divers (3 with pachydrusen, 1 with RPE thickening, and 2 with RPE spikes) representing 12.2%, and 1 eye in the control group (1.5%) showing an RPE spike (Figures 2(a) and 2(b)). RPE abnormalities exhibited a statistically significant rise in both the scuba diver and deep diver groups (*p*=0.01).

The subfoveal choroidal thickness (SFCT) was found to be statistically significantly thicker in the deep divers than in the scuba divers (*p*=0.046) (Table 5) (Figure 2(c)). The mean CT was the thickest in the deep divers (405.32 ± 81.58 μm), followed by the control group (377.26 ± 77.84 μm), and the thinnest in the scuba group (362.32 ± 77.96 μm), but the difference between the groups was statistically insignificant (*p*=0.07).

There was no significant difference in the peripapillary RNFL thicknesses in the four quadrants between the groups (*p* > 0.05) (Table 5).

## 4. Discussion

To the best of our knowledge, this is the first study evaluating the long-term effects of diving on the macula, peripapillary nerve fiber layer, and peripheral retina. It is also the first to compare deep divers and scuba divers. The present study showed significantly increased SFCT and RPE thickening in the 3 mm ring of the ETDRS grid in the deep diver group. These findings show similarities with pachychoroid spectrum disorders.

Pachychoroid disease has been recently described and refers to a group of pathological lesions associated with the choroid. Characteristic features include choroidal hyperpermeability, congestion, and dilated choroidal vessels. The term pachychoroid spectrum encompasses conditions such as CSR, pachychoroid pigment epitheliopathy, pachychoroid neovasculopathy, polypoidal choroidal vasculopathy, and peripapillary pachychoroid syndrome [14–16]. Pachychoroid disease primarily causes abnormalities in the retina, particularly in the RPE and photoreceptor outer segments, which manifest as mottling of the RPE, small RPE detachments, hyper-reflective RPE spikes, and RPE thickening [17].

In a study evaluating ophthalmic parameters after scuba diving, SFCT decreased 30 min after the dive but returned to predive values after 60 min [10]. This was explained by the reduced dilation of the choroidal vascular system, similar to the mechanism of reduced flow-mediated dilation. Endothelial dysfunction and insufficient relaxation of the choroidal circulation were considered responsible for the transient SFCT decrease [10]. In the present study, SFCT was significantly thicker in the deep diver group than in both the scuba diver and control groups. Deleu et al. [10] studied SFCT to explain the common history of CSR in scuba divers, but they only observed a transient decrease in SFCT. They aimed to find outcomes similar to the results of the present study. The difference in results between the two studies may be due to the focus on transient effects in the Deleu et al. study, whereas the current study evaluates long-term outcomes.

RPE thickening was a notable finding in the present study. This is also one of the features of the pachychoroid spectrum. RPE thickening, RPE elevation, and RPE detachment, along with other pachychoroid features, constitute pachychoroid pigment epitheliopathy [16]. Karacorlu et al. [16] detected RPE thickening in 21.6% of patients with pachychoroid pigment epitheliopathy. In our study, in addition to the statistically significant increase in RPE layer thickness in deep divers, other RPE abnormalities, such as RPE spikes and pachydrusen, were also significantly more frequent in the deep divers. These findings suggest pachychoroid pigment epitheliopathy. In their 1988 study, Polkinghorn et al. [18] reported similar findings using fluorescein angiography (FFA) and associated them with DCS. However, with today's understanding, these findings also appear to be related to the pachychoroid spectrum [18].

The mechanisms causing pachychoroid disease features could be linked to pathophysiological events occurring during diving. There are four potential mechanisms that may lead to the development of these findings in divers. *Venous congestion* is the most likely mechanism. Elevated retrograde venous pressure transmitted to the vortex veins may result in the engorgement of Haller's vessels and, subsequently, the accumulation of subretinal fluid [19]. Ambient pressure increases during descent in direct proportion to depth and decreases during ascent [11]. In the SSDS, the gas pressure inside the helmet increases with depth and may exert positive pressure on the globe [20]. This pressure can cause venous congestion in the choroidal vessels, contributing to pachychoroid formation.

Another proposed etiology for pachychoroid pathology in divers is *endothelial dysfunction* and the resulting capillary hyperpermeability. Increased oxidative stress and venous bubble formation induce endothelial dysfunction in the hyperbaric and hyperoxic environment of diving [21–23]. Oxidative stress exacerbates endothelial dysfunction by oxidizing nitric oxide (NO) and reducing the availability of endothelium-derived vasodilators like prostacyclin and endothelium-derived hyperpolarizing factor [21]. Venous bubble formation is another contributing factor to endothelial dysfunction in divers. Bubbles form in the body even with proper decompression after scuba diving [5]. Circulating bubbles increase shear stress on the endothelium and activate it, leading to the release of endothelial microparticles and an increase in neutrophils [22, 23]. These microparticles can cause endothelial dysfunction at remote sites [24].

Interleukin-6 (IL-6) is a cytokine that increases both retinal and choroidal hyperpermeability. Elevated IL-6 levels have been observed in the plasma of patients with CSR [25]. NO and its derivatives also increase in plasma during diving [26]. NO synthase is present in Müller cells, RPE, and choroidal blood vessels [27]. NO plays a role in the normal phagocytosis of photoreceptor outer segments, as well as in infectious and ischemic processes [27]. Inhibition of NO synthase has been shown to reduce CT and anterior choroidal blood flow in animal studies [28, 29].

**Figure 3:**
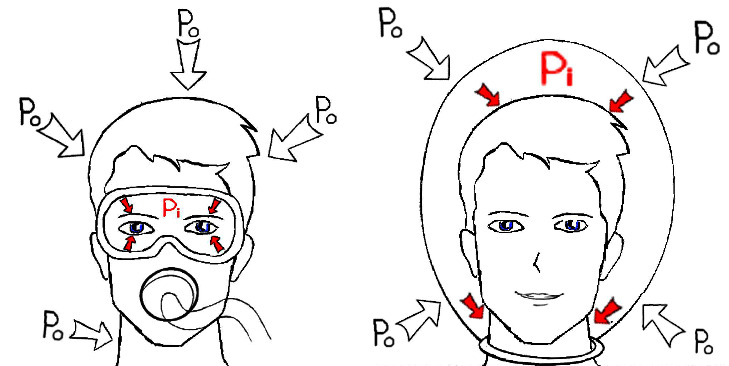
(a) The pressure outside (Po) and inside (Pi) the mask in a scuba diver and (b) the Po and Pi the helmet in a deep diver.

While scuba divers inhale through a separate device from the mouth, they exhale into a half-face mask covering the periorbital area and nose. During exhalation, they attempt to create a small amount of positive pressure inside the mask. This positive pressure applied to the orbit in scuba divers may cause flattening of the globe and reduction of the choroidal layer due to this morphological change (Figure 3(a)). Deep divers, on the other hand, dive with helmets that cover the entire head and face. Therefore, the pressure around the eyes and cranium is equal in deep divers (Figure 3(b)). Our hypothesis is that deep divers experience intracranial and periorbital venous congestion originating from the neck, which results in choroidal thickening.

Although the scuba diver group demonstrated thinning in SFCT, the RPE abnormalities observed in the deep diver group were also present in the scuba group. RPE abnormalities were a common finding in both diver groups. This may indicate that, in addition to venous congestion and pressure imbalances, other pathophysiological mechanisms related to diving are at play.

SFCT was found to be statistically significantly thicker in the deep divers than in the scuba diver group (417.31 ± 74.64 μm in the deep divers; 372.73 ± 72.42 μm in the scuba divers). However, the control group in the current study demonstrated higher values than those reported in other studies for normal SFCT (393.83 ± 76.39 μm in the control group). According to one study, normal SFCT ranged between 250 and 350 μm in healthy subjects [30]. Another study reported a central CT of 308.7 ± 64.5 μm in the Turkish population [31]. The control group in our study consisted of individuals who regularly performed conditioning exercises, unlike the general population. No articles could be found regarding the long-term effects of exercise on SFCT for comparison.

A limitation of the present study is its cross-sectional nature. A longitudinal study evaluating retinal and choroidal changes over time could provide more informative insights.

In conclusion, subfoveal choroid and RPE thickening, which is also observed in pachychoroid pigment epitheliopathy, was identified as a long-term effect of diving in deep divers. The effects of pressure changes on venous circulation and globe morphology may be the primary reasons for changes in RPE and choroid thickness during diving. However, the abnormalities in RPE also suggest the involvement of endothelial dysfunction and other related pathophysiological mechanisms.

## Figures and Tables

**Table 1:** Comparison of the mean age, BCVA, refractive values, and diving time between groups.

	Deep diver group (52 eyes)	Scuba diver group (49 eyes)	Control group (66 eyes)	*p*
Age (year)				
Mean ± SD	34.85 ± 7.39	34.33 ± 7.92	32.79 ± 6.49	0.271^a^
BCVA (Snellen)	1.00	1.00	1.00	—
Refraction (SE)				
Mean ± SD	−0.17 ± 0.29	−0.10 ± 0.17	−0.06 ± 0.22	0.099^b^
Min/max	(−1.00/+0.25)	(−0.50/0.00)	(−0.75/+0.50)	
Diving time (hours)				
Mean ± SD	455.00 ± 318.88	451.67 ± 281.10	—	0.960^c^
Min/max	(80/1300)	(50/1100)		

Abbreviations: BCVA, best-corrected visual acuity; Max, maximum; Min, minimum; SD, standard deviation; SE, spherical equivalent.

^a^One-way ANOVA.

^b^One-way ANOVA (Welch's).

^c^Independent samples *t*-test.

**Table 2:** Thickness of all retinal layers in the central 1 mm of Early Treatment Diabetic Retinopathy Study grid.

Central 1 mm	Deep diver group	Scuba diver group	Control group	*p*
RT	274.19 ± 22.62	268.35 ± 18.24	268.73 ± 17.55	0.226∗
RNFL	12.12 ± 2.24	12.00 ± 2.02	12.02 ± 2.04	0.954∗
GCL	16.23 ± 4.95	15.10 ± 3.64	15.38 ± 3.85	0.360∗
IPL	21.15 ± 3.55	20.35 ± 3.00	20.61 ± 3.32	0.454∗
INL	19.37 ± 5.16	17.67 ± 3.73	17.94 ± 4.28	0.113∗
OPL	22.04 ± 4.90	23.65 ± 4.35	24.38 ± 6.85	0.080∗
ONL	93.65 ± 14.58	93.22 ± 10.68	91.76 ± 10.55	0.663∗
RPE	16.81 ± 1.85	16.33 ± 1.40	16.62 ± 1.66	0.342∗

*Note:* Thicknesses are expressed in micrometers as mean ± standard deviation. *p* < 0.05 shows statistical significance.

Abbreviations: GCL, ganglion cell layer; IPL, inner plexiform layer; ONL, outer nuclear layer; OPL, outer plexiform layer; RNFL, retinal nerve fiber layer thickness; RPE, retinal pigment epithelial layer; RT, retinal thickness.

^∗^One-way ANOVA.

**Table 3:** Thickness of all retinal layers in the inner 3 mm ring of Early Treatment Diabetic Retinopathy Study grid.

Inner 3-mm ring	Deep diver group	Scuba diver group	Control group	*p*
RT	350.00 ± 14.54	347.57 ± 14.43	349.30 ± 13.99	0.679^a^
RNFL	25.38 ± 12.15	22.33 ± 2.06	23.03 ± 8.63	0.181^a^
GCL	53.55 ± 4.36	53.85 ± 4.11	54.43 ± 3.95	0.500^a^
IPL	43.29 ± 3.01	43.15 ± 2.69	43.78 ± 2.53	0.424^a^
INL	41.72 ± 3.27	41.43 ± 3.08	42.24 ± 3.41	0.402^a^
OPL	32.30 ± 2.99	34.08 ± 4.86	32.33 ± 3.48	0.073^a^
ONL	74.38 ± 7.26	72.06 ± 8.16	73.42 ± 7.70	0.318^a^
RPE	14.89 ± 1.39	14.39 ± 1.29	14.32 ± 1.14	D > S 0.049^b^ D > C 0.017^b^

*Note:* Thicknesses are expressed in micrometers as mean ± standard deviation. *p* < 0.05 shows statistical significance.

Abbreviations: C, control group; D, deep diver group; GCL, ganglion cell layer; IPL, inner plexiform layer; ONL, outer nuclear layer; OPL, outer plexiform layer; RNFL, retinal nerve fiber layer thickness; RPE, retinal pigment epithelial layer; RT, retinal thickness; S, scuba diver group.

^a^One-way ANOVA.

^b^One-way ANOVA, post hoc, LSD.

**Table 4:** Thickness of all retinal layers in the outer 6-mm ring of Early Treatment Diabetic Retinopathy Study grid.

Outer 6-mm ring	Deep diver group	Scuba diver group	Control group	*p*
RT	303.76 ± 14.05	303.68 ± 11.56	306.05 ± 12.31	0.511∗
RNFL	35.66 ± 4.82	36.14 ± 3.27	36.41 ± 3.93	0.605∗
GCL	37.26 ± 4.10	37.96 ± 2.84	37.80 ± 3.63	0.581∗
IPL	30.50 ± 3.34	30.72 ± 2.40	30.69 ± 2.78	0.912∗
INL	33.67 ± 2.51	34.29 ± 1.96	34.71 ± 2.76	0.107∗
OPL	27.73 ± 1.98	27.80 ± 2.46	27.49 ± 1.88	0.705∗
ONL	60.17 ± 5.29	58.60 ± 6.79	60.53 ± 5.66	0.204∗
RPE	13.30 ± 1.24	12.84 ± 1.15	12.86 ± 1.05	0.071∗

*Note:* Thicknesses are expressed in micrometers as mean ± standard deviation. *p* < 0.05 shows statistical significance.

Abbreviations: GCL, ganglion cell layer; IPL, inner plexiform layer; ONL, outer nuclear layer; OPL, outer plexiform layer; RNFL, retinal nerve fiber layer thickness; RPE, retinal pigment epithelial layer; RT, retinal thickness.

^∗^One-way ANOVA.

**Table 5:** Peripapillary RNFL and subfoveal choroidal thickness comparisons between groups.

	Deep diver group	Scuba diver group	Control group	*p*
S-RNFL	125.56 ± 13.28	127.98 ± 16.42	126.23 ± 14.17	0.695^a^
I-RNFL	129.28 ± 12.23	131.00 ± 13.80	128.74 ± 18.73	0.719^a^
T-RNFL	77.06 ± 9.53	79.10 ± 10.78	79.08 ± 8.04	0.448^a^
N-RNFL	65.82 ± 7.92	66.90 ± 10.79	67.89 ± 7.51	0.448^a^
SFCT	417.31 ± 74.64	372.73 ± 72.42	393.83 ± 76.39	D > S 0.012^b^

*Note:* Thicknesses are expressed in micrometers as mean ± standard deviation. *p* < 0.05 shows statistical significance.

Abbreviations: D, deep diver group; I-RNFL, inferior RNFL; N-RNFL, nasal RNFL; RNFL, retinal nerve fiber layer; S, scuba diver group; SFCT, subfoveal choroidal thickness; S-RNFL, superior RNFL; T-RNFL, temporal RNFL.

^a^One-way ANOVA.

^b^One-way ANOVA post hoc Tukey HSD.

## Data Availability

The datasets used and/or analyzed during the current study are available from the corresponding author on reasonable request.
